# Mesenchymal Stem Cell Behavior under Microgravity: From Stress Response to a Premature Senescence

**DOI:** 10.3390/ijms24097753

**Published:** 2023-04-24

**Authors:** Renzo Pala, Sara Cruciani, Alessia Manca, Giuseppe Garroni, Mohammed Amine EL Faqir, Veronica Lentini, Giampiero Capobianco, Antonella Pantaleo, Margherita Maioli

**Affiliations:** 1 Department of Biomedical Sciences, University of Sassari, Viale San Pietro 43/B, 07100 Sassari, Italy; 2 Department of Medical, Surgical and Experimental Sciences, Gynecologic and Obstetric Clinic, University of Sassari, 07100 Sassari, Italy; 3 Center for Developmental Biology and Reprogramming (CEDEBIOR), Department of Biomedical Sciences, University of Sassari, Viale San Pietro 43/B, 07100 Sassari, Italy

**Keywords:** mesenchymal stem cells, cell senescence, cellular mechanisms, stress response, microgravity

## Abstract

Mesenchymal stem cells are undifferentiated cells able to acquire different phenotypes under specific stimuli. Wharton’s jelly is a tissue in the umbilical cord that contains mesenchymal stromal cells (MSCs) with a high plasticity and differentiation potential. Their regeneration capability is compromised by cell damage and aging. The main cause of cell damage is oxidative stress coming from an imbalance between oxidant and antioxidant species. Microgravity represents a stressing condition able to induce ROS production, ultimately leading to different subcellular compartment damages. Here, we analyzed molecular programs of stemness (Oct-4; SOX2; Nanog), cell senescence, p19, p21 (WAF1/CIP1), p53, and stress response in WJ-MSCs exposed to microgravity. From our results, we can infer that a simulated microgravity environment is able to influence WJ-MSC behavior by modulating the expression of stress and stemness-related genes, cell proliferation regulators, and both proapoptotic and antiapoptotic genes. Our results suggest a cellular adaptation addressed to survival occurring during the first hours of simulated microgravity, followed by a loss of stemness and proliferation capability, probably related to the appearance of a molecular program of senescence.

## 1. Introduction

Human mesenchymal stem cells are multipotent elements able to restore tissue function after injury [1]. Wharton’s jelly is a tissue within the umbilical cord and represents the primary connective tissue, containing mesenchymal stromal cells (MSCs), which were first described by Thomas Wharton in 1656 [2]. Stem cells derived from WJ-MSCs represent a valuable model of multipotent stem cells and can be easily obtained from “wasting materials” without ethical problems [2,3,4,5,6]. As compared to mesenchymal stem cells (MSCs) derived from bone marrow or adipose tissue, these cells exhibit a younger phenotype and a related higher plasticity [7,8]. Indeed, the activities and functions of tissue-derived MSCs decline with aging, causing significant complications for MSC-based treatments in regenerative medicine [9,10]. In addition, WJ-MSCs maintain their shape for a long period of time during passages in vitro [11,12] together with their immunoprivileged status even after differentiating into several lineages. Moreover, WJ-MSCs have previously been extensively investigated in several clinical trials and research initiatives with impressive results [13,14]. Stem cell pluripotency is a key feature controlled by several regulatory genes, including octamer-binding transcription factor 4 (Oct-4), SOX2 and NANOG, which have been widely used in vitro for iPS generation [15,16]. The stem cell genes Oct-4, SOX2 and NANOG form a triad of transcription factors crucial for maintaining stem cell capabilities by activating genes in the self-renewal program and inhibiting genes involved in differentiation, thus regulating thousands of loci in the genome to maintain an undifferentiated state. It has been largely demonstrated that these genes are gradually downregulated during aging and the senescence of stem cells, thus affecting the ability of stem cells to adopt a specific cellular phenotype [17,18,19]. Cells are continuously exposed to reactive oxygen species (ROS), which are normally generated by cell metabolism and environmental stressors such as X-rays, UV rays, pollution and xenobiotics. Within this context, the main cause of cell damage is oxidative stress caused by an imbalance between oxidizing and antioxidant species [20,21,22]. This condition can be ameliorated by cellular detoxification mechanisms that physiologically protect cells. Cells can react to a stressor, including high levels of ROS, by employing the longevity factor and deacetylase SIRT1. The stress inducibility of SIRT1 plays a role in cytoprotection and cancer, as do HSP60, HSP70 and NOX4 [23]. Within the different extreme environments able to induce oxidative stress, microgravity represents a novel emerging situation, affecting different systems and mechanisms such as repair, replication, transcription, and protein expression, leading to an increase in ROS and consequently causing damage to cellular lipid membranes, mitochondria, proteins and DNA [24,25]. It has been observed that the microgravity environment leads to changes in the transcriptome and cytoskeleton, resulting in both short- and long-term morphological cellular changes [26]. Among the cellular mechanisms activated during stress exposure, apoptosis cytochrome C (Cyt C) can be included. In particular, Cyt C, although usually involved in the respiratory chain, can escape from the mitochondrion when the organelle is damaged or when it receives specific instructions. Once released from its usual context, Cyt C can be recruited and contribute to the apoptotic dismantling of the cell [27]. Within this context, identifying novel tools able to mimic microgravity features on Earth is an area of growing interest [28]. To reproduce microgravity conditions, a g-force between 10^−3^ and 10^−6^ must be achieved, resulting in a weightless environment [29]. In order to reproduce these conditions, machines validated by international space agencies have been used, namely random positioning machines (RPM) and 3D clinostats [30]. The purpose of these devices is to generate a rapid rotation along three axes, leading to a condition of weightlessness [31,32]. In the present paper, the microgravity environment was achieved using a vectorless uniaxial horizontal clinostat. The clinostat-based model system is a ground-based method to achieve vector-averaged gravity reduction on cell cultures. Its function is to randomize the orientation of the gravity vector, which is why it has been used to generate simulated microgravity environments for more than 10 years [33,34]. In the present study, we explored the effect of microgravity on defined WJ-MSC behaviors such as cell proliferation, cell senescence and stemness properties. In particular, we analyzed the molecular program of senescence by examining the expression of the following genes: p16, p21, p19 and p53 and of the stemness genes Oct-4, SOX2 and NANOG. Additionally, we analyzed cellular response to stressing conditions, evaluating SIRT1, HSP60, HSP70 and NOX4, the cytoskeleton markers β-Actin and β-Tubulin and the apoptosis regulators BAX and BCL2, together with cytochrome C.

## 2. Results

### 2.1. Microgravity Affects Stemness Gene Expression

**Figure 1:**
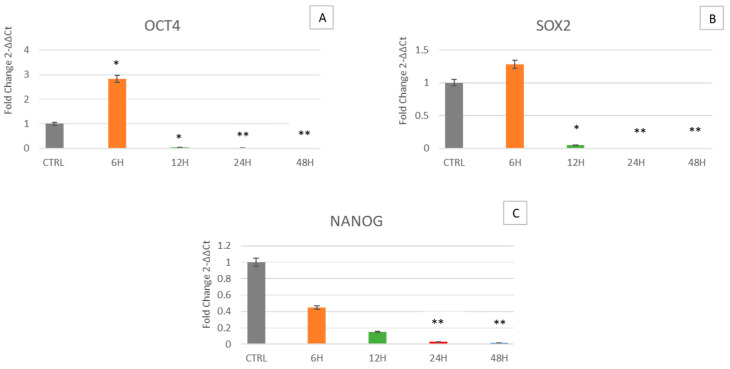
Expression of stemness genes. The expression of the stemness-related genes Oct-4 (**A**), SOX2 (**B**) and NANOG (**C**) was assessed in WJ-MSCs cultured under simulated microgravity (µg) for 6 h-µg (orange bars), 12 h-µg (green bars), 24 h-µg (red bars) or 48 h-µg (blue bars). The mRNA levels for each gene were normalized against glyceraldehyde-3-phosphate-dehydrogenase (GAPDH) and were expressed as fold of change (2^−ΔΔCt^) in mRNA levels observed in controls WJ-MSCs (Ctrl). Controls WJ-MSCs (gray bars) are defined as 1 (mean ± SD; *n* = 6). Data are expressed as mean ± SD referenced to control (* *p* ≤ 0.05), (** *p* ≤ 0.01).

Figure 1 shows that in cells cultured for 6 h in the clinostat, Oct-4 is overexpressed, while SOX2 and NANOG show no significant differences as compared to control cells not exposed to microgravity. After 12, 24 and 48 h of culturing in the clinostat, Oct-4, SOX2 and NANOG expression were significantly downregulated, being only faintly detectable.

### 2.2. Simulated Microgravity Affects a Molecular Pattern of Stress Response 

**Figure 2:**
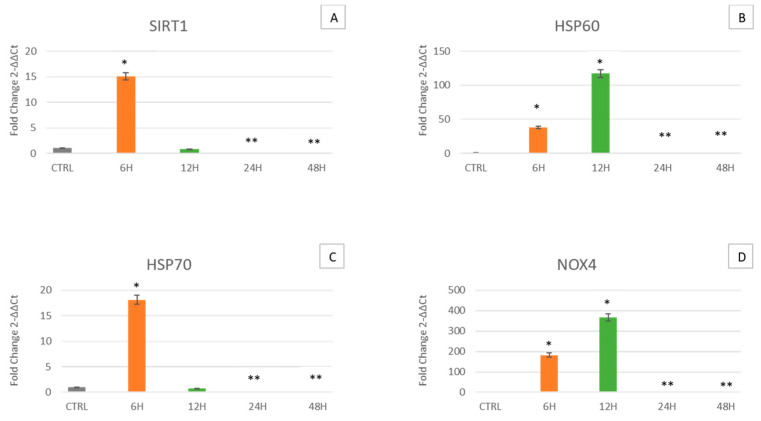
Analysis of sirtuins, heat shock protein and NADPH oxidase. Expression of SIRT1 (**A**), HSP60 (**B**), HSP70 (**C**), and NOX4 (**D**) was assessed in WJ-MSCs cultured under simulated microgravity for microgravity (µg) for 6 h-µg (orange bars), 12 h-µg (green bars), 24 h-µg (red bars) or 48 h-µg (blue bars). The mRNA levels for each gene were normalized against glyceraldehyde-3-phosphate dehydrogenase (GAPDH) and were expressed as fold of change (2^−ΔΔCt^) in the mRNA levels observed in controls WJ-MSCs (Ctrl). Controls WJ-MSCs (gray bars) are defined as 1 (mean ± SD; *n* = 6). Data are expressed as mean ± SD referred to control (* *p* ≤ 0.05), (** *p* ≤ 0.01).

Figure 2 shows that in cells cultured for 6 h in the clinostat, SIRT1 is overexpressed whereas after 12 h, the expression was similar to what was observed in control cells (Figure 2A). In cells cultured for 24 and 48 h, the expression levels gradually decreased, becoming similar to those observed in control cells. The same trend was observed in HSP70 (Figure 2C). Meanwhile, the HSP60 (Figure 2B) and NOX4 (Figure 2D) genes are overexpressed in cells cultured under microgravity for 6 and 12 h compared to controls, but their expression gradually decreases after 24 h of culturing.

### 2.3. Simulated Microgravity Activates a Molecular Program of Cell Senescence 

**Figure 3:**
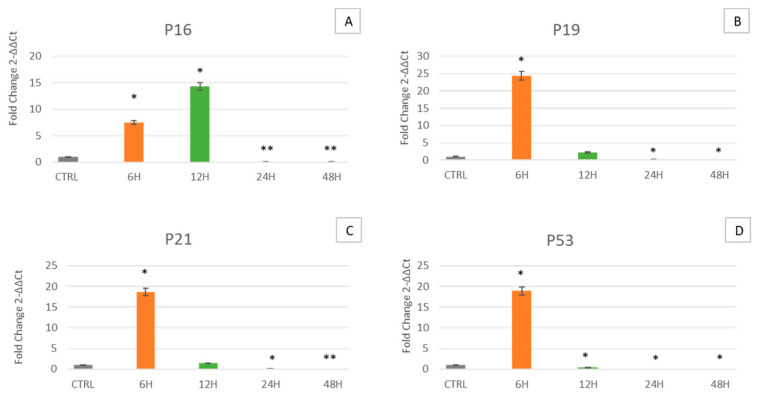
Expression of specific senescence-related markers p16, p19ARF, p21 and p53. Expression of p16 (**A**), p19ARF (**B**), p21 (**C**) and p53 (**D**) was assessed in WJ-MSCs cultured under simulated microgravity (µg) for 6 h-µg (orange bars), 12 h-µg (green bars), 24 h-µg (red bars) or 48 h-µg (blue bars). The mRNA levels for each gene were normalized against glyceraldehyde-3-phosphate dehydrogenase (GAPDH) and were expressed as fold of change (2^−ΔΔCt^) in the mRNA levels observed in controls WJ-MSCs (Ctrl). Controls WJ-MSCs (gray bars) are defined as 1 (mean ± SD; *n* = 6). Data are expressed as mean ± SD referred to control (* *p* ≤ 0.05), (** *p* ≤ 0.01).

Figure 3 shows that in cells cultured for 6 h in the clinostat, p16 (Figure 3A), p19 (Figure 3B), p21 (Figure 3C) and p53 (Figure 3D) are overexpressed as compared to control cells. Nevertheless, in cells cultured for 12, 24 and 48 h, the expression levels of p19 (Figure 3B), p21 (Figure 3C) and p53 (Figure 3D) gradually decrease, becoming similar to those observed in control cells (grey bars). Nevertheless, p16 follows the same trend after 24 and 48 h, being overexpressed in cells cultured under microgravity compared to control cells after 12 h. 

### 2.4. Simulated mMicrogravity Modulates the Expression of Bax and Bcl2 Apoptotis Related Genes

**Figure 4:**
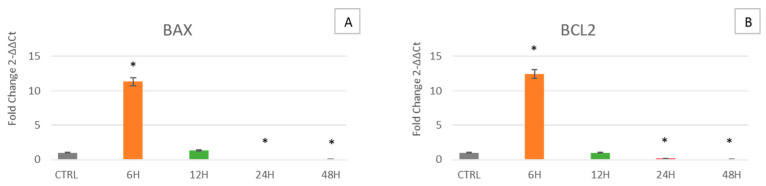
Expression of the apoptotic markers BAX and Bcl-2. Expression of BAX (**A**) and Bcl-2 (**B**) was assessed in WJ-MSCs cultured under simulated microgravity for microgravity (µg) for 6 h-µg (orange bars), 12 h-µg (green bars), 24 h-µg (red bars) or 48 h-µg (blue bars). The mRNA levels for each gene were normalized against glyceraldehyde-3-phosphate dehydrogenase (GAPDH) and were expressed as fold of change (2^−ΔΔCt^) in the mRNA levels observed in controls WJ-MSCs (Ctrl). Controls WJ-MSCs (gray bars) are defined as 1 (mean ± SD; *n* = 6). Data are expressed as mean ± SD referred to control (* *p* ≤ 0.05).

Figure 4 shows that in cells cultured for 6 h in the clinostat, BAX is overexpressed as compared to control cells. However, in cells cultured for 12, 24 and 48 h, the expression levels gradually decrease, becoming similar to those observed in control cells (Figure 4A). The same trend was observed for Bcl-2 (Figure 4B).

### 2.5. Effects of Simulated Microgravity on the Cytoskeleton

**Figure 5:**
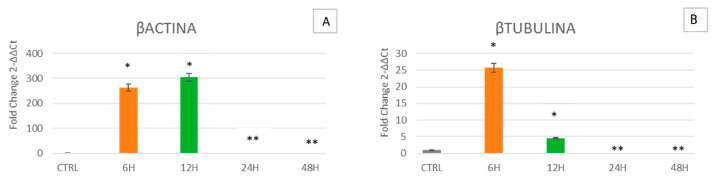
Expression of the principal cytoskeleton markers β-Actin and β-Tubulin. Expression of β-Actin (**A**) and β-Tubulin (**B**) was assessed in WJ-MSCs cultured under simulated microgravity for microgravity (µg) for 6 h-µg (orange bars), 12 h-µg (green bars), 24 h-µg (red bars) or 48 h-µg (blue bars). The mRNA levels for each gene were normalized against glyceraldehyde-3-phosphate dehydrogenase (GAPDH) and were expressed as fold of change (2^−ΔΔCt^) in the mRNA levels observed in controls WJ-MSCs (Ctrl). Controls WJ-MSCs (gray bars) are defined as 1 (mean ± SD; *n* = 6). Data are expressed as mean ± SD referred to control (* *p* ≤ 0.05), (** *p* ≤ 0.01).

Figure 5 shows that in cells cultured for 6 h in the clinostat, β-Tubulin is overexpressed as compared to control cells. Meanwhile, in cells cultured for 12, 24 and 48 h, the expression levels gradually decrease, becoming similar to those observed in control cells (Figure 5B). Meanwhile, β-Actin (Figure 5A) follows the same trend after 24 and 48 h of culturing, while it is overexpressed after 12 h in cells cultured under microgravity as compared to controls.

### 2.6. Expression of Cytochrome C under Microgravity 

**Figure 6:**
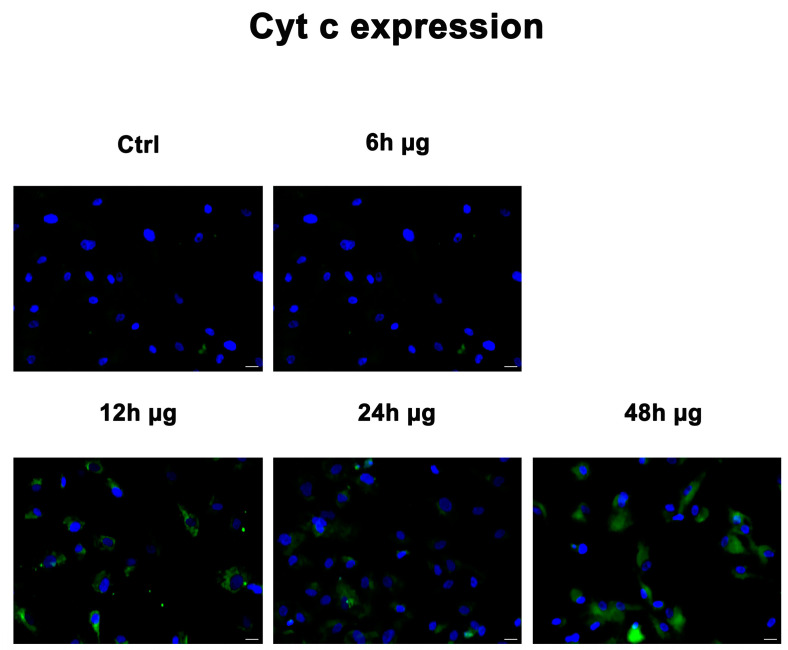
Analysis of Cyt C. Immunofluorescence analysis of Cyt C (green) was assessed in controls WJ-MSCs (Ctrl), and WJ-MSCs incubated from 6 to 48 h under microgravity conditions (µg). The figures are representative of different independent experiments. Nuclei are labelled with 4,6-diamidino-2-phenylindole (DAPI, blue). Magnification 40×. Scale bars: 40 µm.

Immunofluorescence images show the expression of cytochrome C (Cyt C) in WJ-MSCs exposed to microgravity conditions. After 6 h of exposure, the expression levels of Cyt C were similar to controls. The expression levels of Cyt C were increased in cells exposed to simulated microgravity (μg) starting from 12 h onward as compared to control cells (Ctrl) (Figure 6).

### 2.7. Morphological Analysis of WJ-MSCs Exposed to Microgravity

**Figure 7:**
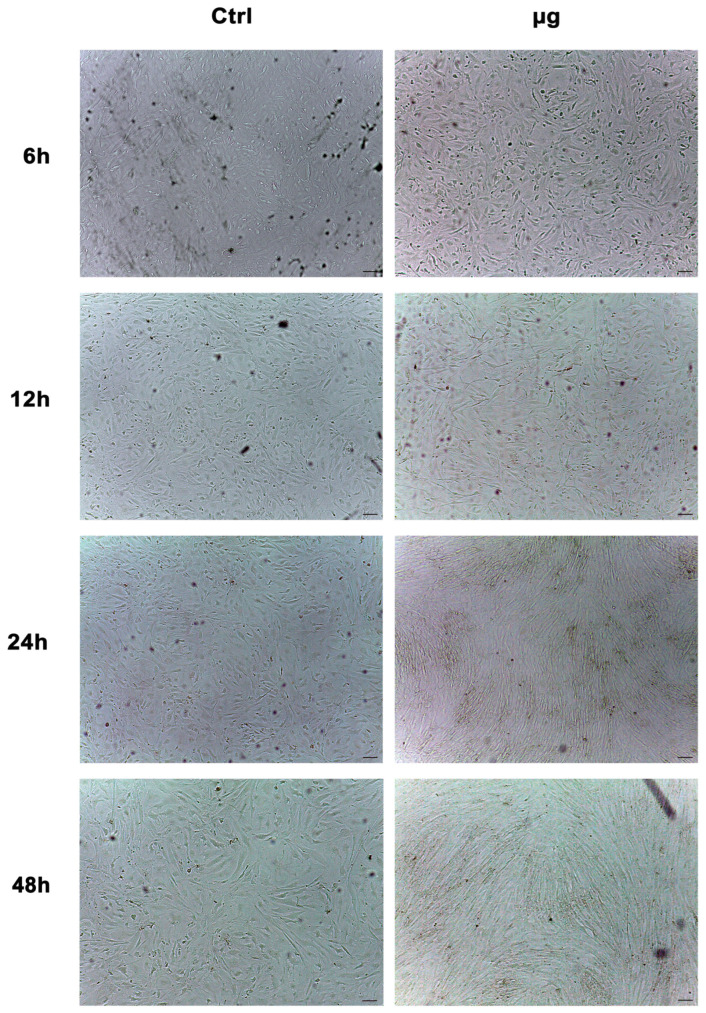
Optical microscope analysis of WJ-MSC morphology after exposure to microgravity (µg). Figure shows morphological changes in cells treated for 6, 12, 24 and 48 h in µg as compared to controls (Ctrl). Scale bar = 100 µm.

Figure 7 shows WJ-MSC morphology after exposure to microgravity. Cells were evaluated with optical microscopy (Leica, Nussloch, Germany) after 6, 12, 24 and 48 h in µg, in accordance with the above-described conditions. For each time point, we did not observe changes in the morphology of the cells exposed to µg as compared to controls (Ctrl).

### 2.8. Evaluation of Protein Expression

**Figure 8:**
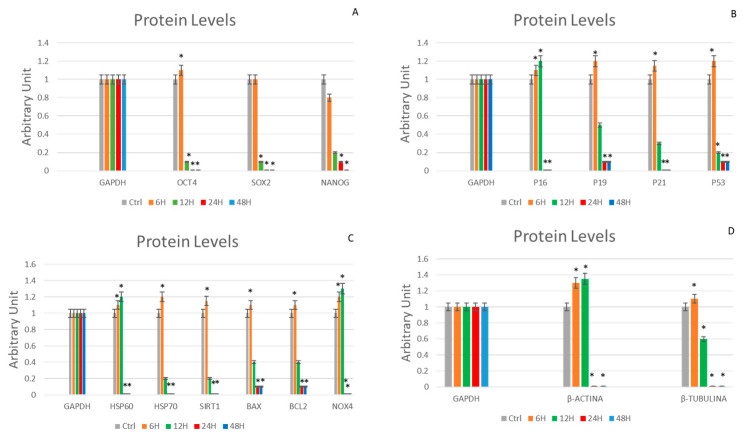
Western blot analysis. Levels of Oct-4, SOX2 and NANOG proteins shown in arbitrary units (**A**). (**B**): levels of p16, p19, p21 and p53 protein shown in arbitrary units. (**C**): levels of Hsp60, Hsp70, Sirt1, Bax, Bcl2 and Nox4 proteins shown in arbitrary units. (**D**): levels of β-Actin and β-Tubulin protein shown in arbitrary units was assessed in WJ-MSCs cultured under simulated microgravity (µg) for 6 h-µg (orange bars), 12 h-µg (green bars), 24 h-µg (red bars) or 48 h-µg (blue bars). The protein levels for each gene were compared against glyceraldehyde-3-phosphate-dehydrogenase (GAPDH). Controls WJ-MSCs (gray bars) are defined as 1 (mean ± SD; *n* = 6). Data are expressed as mean ± SD referenced to control (* *p* ≤ 0.05), (** *p* ≤ 0.01).

Figure 8 shows a western blot analysis of cells cultured for 6, 12, 24 and 48 h under microgravity expressed in arbitrary unit. We can confirm the same trend observed in gene expression analysis. 

## 3. Discussion

Simulated microgravity mimics a space environment, allowing us to unravel cellular behavior and analyze different mechanisms able to face this stressing condition. Here we decided to examine the effect of this condition on WJ-MSCs, a particular type of mesenchymal stem cell, that for their extremely young phenotype exhibit high plasticity and differentiation potential, being more sensitive to any kind of external stimulus. Specifically, WJ-MSCs were cultured for 6, 12, 24 and 48 h under simulated microgravity while control WJ-MSCs were placed in a static bar at 1 g to undergo the same vibrations as the samples under microgravity conditions. The gene expression analysis of the stemness-related genes demonstrates that simulated microgravity significantly affects the differentiation potential of WJ-MSCs (Figure 1). In particular, after 6 h of clinostat treatment, Oct-4 is first overexpressed, while SOX2 and NANOG show no significant differences. However, after longer culturing under microgravity, Oct-4, SOX2 and NANOG gene expression is inhibited. The early observed Oct-4 overexpression can drive a metabolic reprogramming of cells, redirecting glucose catabolism to the glycolysis pathway and acting as a tentative mechanism to counteract senescence [35]. After 12, 24 and 48 h of clinostat exposure, Oct-4, SOX2 and NANOG were downregulated, indicating that microgravity, acts mainly on Oct-4 in promoting cellular senescence processes [36]. Analysis of the mRNA levels of p16, p19, p21 (WAF1/CIP1) and p53 involved in cell cycle arrest and in cell senescence showed that exposure to μg can, at the beginning, induce the expression of p16, p19, p21 and p53 (Figure 3, orange bars). However, after longer periods of exposure to μg (12, 24 and 48 h), a significant overexpression of p16 could be observed while downregulating after 24 and 48 h of culturing under μg, as compared to controls. Meanwhile, after longer periods of exposure to μg (12, 24 and 48 h), a significant downregulation of p19, p21 (WAF1/CIP1) and p53 genes could be observed as compared to controls. A number of regulatory proteins have been suggested to be signals that induce senescence or mediate cell entry into senescence. p16 accumulates as cells undergo increasing numbers of cell divisions and approach senescence [37]. Inactivation of p19Arf accelerates sarcopenia and fat loss, suggesting that its induction suppresses cellular stress that places cells in a state of senescence [38]. p19Arf acts at the nucleolus, where, by binding to other factors, it activates ribosome biogenesis and initiates the p53-dependent processes of senescence and apoptosis [39,40,41]. There is a mild senescent cell state which allows cells to resume proliferation after p53 inactivation and a deep senescent state that is irreversible [42]. Therefore, considering our results, we can assume that WJ-MSCs exposed to microgravity undergo a mild senescence state, also partially losing stemness features. Within this context, other authors previously reported that stem cells in the senescent state express modified features, interfering with their stemness and regenerative potential [43]. The impact of individual p53 target genes on senescence is highly intricate. Here, we focused on the role of p16, p19, p21 and p53, a target gene involved in cell cycle arrest and cellular senescence [44,45]. In addition to revealing that p53 is the critical mediator of p19Arf-induced antiaging responses, p21 has been identified as a key downstream target gene of p53, through which the protective function of p53 is expressed. Stressogenic processes generate an aging response that is balanced by p53 and involves single or multiple targets of p53, depending on tissue type. In addition, p53 is associated with the activation of apoptosis through the apoptotic proteins BAX and Bcl-2 [46]. Interestingly, here we show that under microgravity, the expression of both BAX and Bcl-2, a pro- and anti-apoptotic gene respectively, are induced after a short period of culturing, being downregulated after longer periods of exposure to microgravity. Probably these apparently opposite events could have a third player acting upstream and finally rescuing cells from apoptosis. From a cellular perspective, the importance of mitochondria as a primary source of energy has long been well known. Moreover, mitochondria are also important in myriad other cellular processes such as cell cycle control, apoptosis and regulation of cellular metabolism [47]. Interestingly, both mitochondrial dysfunction and cellular senescence are considered key features of aging. The release of Cyt C observed by us (Figure 6) indicates the initiation of regulation of cellular metabolism. Previous studies have shown that high expression of HSPs is associated with longevity and that decreases in their levels correspond to increased protein deterioration during aging, loss of protein quality control, degeneration and cell death [48]. Two of the targets of our study are HSP60 and HSP70, which, in addition to their function as a chaperone for the folding, transport and assembly of newly synthesized polypeptides [49,50], protect cells from a variety of apoptotic stimuli including heat shock, tumor necrosis factor, growth factor withdrawal, oxidative stress, chemotherapeutic agents and radiation [51,52,53,54,55]. Intracellular HSP60 and HSP70 exhibit cytoprotective, anti-apoptotic and anti-inflammatory functions. Under these conditions, HSP70 prevents caspase-3 activation [56,57]. Despite recent advances, the anti-apoptotic mechanism of HSP70 is still controversial [56,58]. In fact, no correlation between Cyt C release and apoptosis has been reported, since Cyt C can be released even in viable cells [59,60], as inferable by the morphological analysis performed (Figure 7), whereas cells can undergo apoptosis without releasing Cyt C [59,60]. Our results fit perfectly with the well-known capability of HSP60 and HSP70 to inhibit apoptosis downstream of Cyt C release but upstream of caspase-3 cleavage. In fact, we observe an increase in HSP60 and HSP70 gene expression along with an increase in Cyt C detection at the cytoplasmic level (Figure 6). SIRT1 has also been shown to play an important role in determining lifespan and stress resistance [61,62,63]. It has been found that lower circulation levels of SIRT1 in the sera of elderly people are a distinctive marker of cellular senescence [64]. SIRT1 activity declines very rapidly in senescence and a decrease in NAD+ cofactor levels is believed to be the main reason for the decline in SIRT1 activity during aging. SIRT1 is expressed in many tissues and is involved in many types of age-related disease progression. SIRT1 activation significantly arrests the expression of p21 by mitigating the processes of senescence [65]. Here we show that SIRT1 is induced early in microgravity (Figure 4) and then downregulated, being able to counteract the expression of the p21 gene, which is ultimately downregulated (Figure 2). NOX4 protects the vascular system from inflammatory stress. One study found that NOX4 facilitates some beneficial adaptive responses to exercise mediated by ROS. Furthermore, NOX4 downregulation of skeletal muscle during aging and obesity has been shown to contribute to the development of insulin resistance and may promote oxidative stress. [66]. In space, the force of gravity is reduced, resulting in microgravity (µg). Although human cells do not have a gravity sensor, they can still sense µg through the cytoskeleton [67]. We observed how the cells’ response to microgravity (µg) resulted in β-actin gene overexpression after 6 and 12 h of treatment while its downregulation was observed after 24 and 48 h. At the same time, comparing cells cultured under μg to controls, we observed that β-tubulin was overexpressed after 6 h of treatment while being downregulated after 12, 24 and 48 h. Our data suggest that microgravity is able at first to promote cytoskeletal adaptation, which then decreases at later hours, enfeebling the cellular scaffold [68]. On the whole, all the genes analyzed here show the same trend, being upregulated in cells exposed for 6 h to microgravity and then downregulated after longer periods. According to the present results, WJ-MSCs exposed to microgravity have a rapid adaptive stress response after 6 h, leading to increased expression of genes related to stemness, stress response and apoptosis/senescence. After the rapid adaptive response, strong cellular adaptation occurs, leading to gene downregulation and to senescence. In fact, the observed Cyt C release and metabolic cellular adaptation are a trigger able to drive cells toward senescence processes.

## 4. Materials and Methods

### 4.1. WJ-MSC Isolation and Culture

The study included umbilical cords (*n* = 6) retrieved from healthy full-term women between 25 and 35 years old, recruited according to the following criteria: spontaneous birth and donors free from drugs, smoking and diseases. The samples were collected after natural childbirths at the Gynecologic and Obstetric Clinic of the University of Sassari. The patients gave written informed consent according to the approval of this study by the Ethics Committee (Ethical Clearance N. 0021565/2018, 22 March 2018, Commissione Etica CNR). All the experiments were performed twice (in three technical replicates), separately for each of the 6 samples. The umbilical cords were collected in phosphate-buffered saline (PBS) supplemented with 200 U/mL penicillin (Euroclone, Milano, Italy), 200 mg/mL streptomycin (Euroclone, Milano, Italy) and 4 mg/mL amphotericin B (Gibco Life Technologies) prior to storage at 4 °C for further WJ-MSC isolation. Tissues were dissected into small pieces and then digested with collagenase type I (2 mg/mL) (Sigma Aldrich, Schnelldorf, Germany) at 37 °C for 1 h with agitation. After neutralization of the enzyme with 20% fetal bovine serum (FBS) (Life Technologies, Grand Island, NY, USA) and filtering (70 μm cell strainer) (Euroclone, Milano, Italy), samples were centrifuged at 600× *g* for 10 min and cultured in a basic medium (BM), Dulbecco’s modified Eagle’s Medium (DMEM) (Life Technologies, Grand Island, NY, USA) supplemented with 20% fetal bovine serum (FBS) (Life Technologies, Grand Island, NY, USA), 200 mM L-glutamine (Euroclone, Milano, Italy) and 200 U/mL penicillin–0.1 mg/mL streptomycin (Euroclone, Milano, Italy), then cultured in T25 flasks at 37 °C with 5% CO_2_ and saturated humidity for 10–14 days [64]. The culture medium was changed every 3 days. When cells reached 80–90% confluence, they were harvested using 0.25% Trypsin EDTA (Euroclone, Milano, Italy), counted and transferred into new flasks. WJ-MSCs were immunomagnetically sorted for c/kit using a monoclonal anti-c/kit (CD117) antibody (Miltenyi Biotech, Minneapolis, MN, USA) directly conjugated to microbeads (Miltenyi Biotech). The WJ-MSCs used in this study were characterized by flow cytometry as previously described [69]. For each treatment, after counting with the Tripan Blue and LUNA-II™ Automated Cell Counter, 5 × 10^5^ cells/T25 flask were seeded, treated and then used for total RNA extraction.

### 4.2. Microgravity Simulation

To test whether Wharton’s jellies (WJ-MSCs) can be affected by microgravity conditions, experiments were performed using a 3D random positioning machine (RPM, Fokker Space, Netherlands) at the laboratory of the Department of Biomedical Sciences, University of Sassari, Sardinia, Italy. The 3D RPM is a microweight (microgravity) simulator based on the gravity vector-averaging principle, built by Dutch Space. The 3D RPM consists of two perpendicular frames that rotate independently. The direction of the gravity vector is constantly changed so that the gravity-vector-averaging simulates a microgravity environment. The 3D RPM provides a simulated microgravity of less than 10^−3^ g. The dimensions of the 3D RPM are 1000 × 800 × 1000 mm (length × width × height). The RPM 3D is connected to a computer, and, through specific software, the mode and speed of rotation were selected. Random Walk mode at 80 degrees/s (rpm) was chosen. To simulate the effect of all operating conditions, the following procedure was adopted. The T12 flasks and chamber slides were carefully filled with WJ-MSCs and filled with basic medium (BM), Dulbecco’s modified Eagle’s Medium (DMEM) (Life Technologies Grand Island, NY, USA) supplemented with 20% fetal bovine serum (FBS) (Life Technologies, Grand Island, NY, USA), 200 mM L-glutamine (Euroclone, Milano, Italy) and 200 U/mL penicillin–0.1 mg/mL streptomycin (Euroclone, Milan, Italy) without air bubbles to avoid fluid shear, in a dedicated room at 37 °C. Controls were placed in the static bar at 1 g to undergo the same vibration as the sample under µg conditions. Different time points (6–12–24–48 h) were set. Then WJ-MSCs present in the flasks were detached using 0.25% Trypsin EDTA (Euroclone, Milan, Italy), centrifuged and stored at −20 °C until use for further characterization. For chamber slides, the samples were fixed in paraformaldehyde (Sigma-Aldrich Chemie GmbH, Schnelldorf, Germany).

### 4.3. RNA Extraction and Quantitative Polymerase Chain Reaction

After treatment, total RNA was isolated with the reagent TRIzol^®^ and quantified by measuring absorbance at 260/280 nm (NanoDrop 2000, Thermo Fisher Scientific ND8008 spectrophotometer, Thermo Fisher Scientific, Waltham, MA, USA). Approximately 1 µg of total RNA was transcribed into reverse cDNA with the SuperScript^®^ VILO™ cDNA Synthesis Kit (Life Technologies, Grand Island, NY, USA). Quantitative polymerase chain reactions were carried out with a CFX thermal cycler (Bio-Rad, Boston Industries, Hercules, CA, USA), incubated under standard qRT-PCR conditions (50 °C for 2 min, 95 °C for 2 min, and then cycles to 95 °C for 15 s, 55–59 °C for 30 s, and 60 °C for 1 min, for 40 cycles), according to the qRT-PCR protocol specified in the Quantitative PCR Master Mix with Power SYBR^®^ Green. For each reaction, 0.1 µM of each primer and 3 µL of cDNA generated from 1 μg of total RNA were mixed in 25 µL volumes and added. The Ct values of the targets were normalized against GAPDH [54,55,56], which was considered the reference gene, while the gene levels of the stem cells were expressed as fold change (2^−∆∆Ct^) from the gene levels observed when the stem cells had reached 80% confluence before starting treatment. Each experiment included a control with distilled water. qRT-PCR analysis was performed on the following set of genes: Oct-4, SOX2, NANOG, SIRT1, p21 (WAF1/CIP1), p19 (ARF), p53, BAX and Bcl-2. All primers used (Invitrogen) and are described in Table 1.

### 4.4. Immunofluorescence Analysis

WJ-MSCs (2.0  × 10^4^ cells/ chamber slides) were incubated from 6 to 48 h under microgravity conditions (µg) and then fixed with 4% paraformaldehyde (Sigma Aldrich Chemie GmbH, Germany) for 30 min at room temperature. Control cells (Ctrl) were cultured under terrestrial (1 g) conditions. After fixation, cells were permeabilized using 0.1% Triton X-100 (Thermo Fisher Scientific, Grand Island, NY, USA)-PBS and then washed in PBS three times for 5 min. After washing, WJ-MSCs were incubated with 3% Bovine Serum Albumin (BSA)—0.1% Triton X-100 in PBS (Thermo Fisher Scientific, Grand Island, NY, USA) for 30 min and then exposed overnight at 4 °C to the primary monoclonal mouse cytochrome C (Cell Signalling, Danvers, MA, USA) primary antibody. After three washing steps in PBS for 5 min, cells were stained at 37  °C for 1 h in the dark with fluorescence-conjugated goat and mouse IgG secondary antibody (Life Technologies, Grand Island, NY, USA). Nuclei were labelled with 1 µg/mL 4,6-diamidino-2-phenylindole (DAPI) (Thermo Fisher Scientific, Grand Island, NY, USA). All microscopy analyses were performed using a confocal microscope (TCS SP5, Leica, Nussloch, Germany).

### 4.5. Western Blot Analysis

Cells were cultured in the above-described conditions for 6, 12, 24 and 48 h under microgravity. Protein extraction was performed by RIPA Lysis and Extraction Buffer (Thermo Fisher Scientific, Grand Island, NY, USA; ref. 89,900). For SDS-PAGE analysis, samples were first thawed and then incubated at 95 °C for 5 min. Samples were loaded onto a 10% polyacrylamide gel, subjected to electrophoresis and transferred onto nitrocellulose membranes for western blotting. Nonspecific binding was blocked by incubating membranes overnight in 5% non-fat milk–TBST [25 mM Tris, 140 mM NaCl, 3 mM KCl, 0.5% (*v/v*) Tween-20, pH 8.0] at 4 °C with rocking. Membranes were then probed with anti-p-Tyr (PY99) (1:1000; mouse monoclonal; Santa Cruz Biotechnology # SC-7020, Segrate, Italy) and anti-actin (1:10,000; rabbit polyclonal; Sigma Aldrich #A2103) antibodies in TBST for 1 h at room temperature (RT) with gentle agitation. After washing, the membranes were incubated in secondary antibodies (1:10,000 anti-rabbit IgG, HRP-linked or 1:10,000 mouse IgG, HRP-linked antibody according to the isotype of the primary antibody; Jackson ImmunoResearch Laboratories Inc. #715-035-150(Mouse) or #711-035-152(Rabbit)) for 30–60 min at RT with shaking and subsequently washed with TBST. When needed, a specific anti-phosphotyrosine antibody capable of detecting phosphorylated tyrosine 8 of band 3 was used in place of the nonspecific anti-phosphotyrosine antibody at 1:5000 dilution. The anti-phosphotyrosine 8 antibody was prepared in our lab with the help of Proteintech Inc. (Proteintech; antigen name: Li2760-EC1). Mouse monoclonal anti-band 3 antibody was obtained from Sigma Chemical Co. (#B9277) and used for band 3 staining at 1:10,000 dilution. Proteins were visualized by incubation with chemiluminescent substrate on a ChemiDoc Imaging System using Image Lab software (Bio-Rad) [70].

### 4.6. Statistical Analysis

Statistical analysis was performed using GraphPad Prism 9.0 software (GraphPad, San Diego, CA, USA). For each treatment, two separate experiments with three technical replicates were performed. Two-way analysis-of-variance ANOVA tests with Tukey’s correction and the Wilcoxon signed-rank test were used, assuming a *p* value < 0.05 as statistically significant. We considered * *p* < 0.05, ** *p* < 0.01, *** *p* < 0.001.

## 5. Conclusions

Here we analyze WJ-MSC behavior under simulated microgravity. Our results highlight an early cellular response to stress, followed by an adaptive senescence state, stabilized by a complex interplay between all the different factors examined. Our data demonstrate how microgravity conditions can lead not only to changes in the expression of key genes involved in differentiation, stress and senescence processes, but also to cytoskeleton rearrangement. Further studies are needed in order to better understand which cellular mechanisms are involved in cellular response to microgravity. Nevertheless, our findings represent interesting preliminary data able to better define stem cell destination and tissue regeneration in a microgravity environment and pave the way for future defensive strategies. Our results, even if preliminary, add new insight in the field, unraveling stem cell regenerative behavior for future space missions.

## Figures and Tables

**Table 1:** Primer sequences.

Primer Name	Forward	Reverse
Oct-4	GAGGAGTCCCAGGCAATCAA	CATCGGCCTGTGTATATCCC
SOX2	CCGTTCATGTAGGTCTCGGAGCTG	CAACGGCAGCTACAGCTAGATGC
NANOG	CATGAGTGTGGATCCAGCT	CCTGAATAAGCAGATCCAT
P19	GCCTTCGGCTGACTGGCTGG	TCGTCCTCCAGAGTCGCCCG
P21 (WAF1/CIP)	CAAAGGCCCGCTCTACATCTT	AGGAACCTCCATTCACCCGA
P53	CAAGCAATGGATGATTTGATGCT	TGGGTCTTCAGTGAACCATTGT
BAX	TGCTTCAGGGTTTCATCCAG	GGCGGCAATCATCCTCTG
Bcl-2	AGGATTGTGGCCTTCTTTGA	ACAGTTCCACAAAGGCATCC
HSP70	CACAGCGACGTAGCAGCTCT	ATGTCGGTGGTGGGCATAGA
SIRT1	CATTTCCATGGCGCTGAGG	TGCTGGTGGAACAATTCCTGT
GAPDH	GAGTCAACGGATTTGGTCGT	GACAAGCTTCCCGTTCTCAG
P16	CTCGTGCTGATGCTACTGAGGA	GGTCGGCGCAGTTGGGCTCC
HSP60	GGGCATCTGTAACTCTGTCTT	TAAAAGGAAAAGGTGACAAGG
NOX4	GATGACTGGAAACCATACAAG	TAAAAGTTTCCACCGAGGACG
β-Actin	CACCATTGGCAATGAGCGGTTC	AGGTCTTTGCGGATGTCCACGT
β-Tubulin	CTGGACCGCATCTCTGTGTACT	GCCAAAAGGACCTGAGCGAACA

## Data Availability

Data are contained within the article.
