# Correction: Pala et al. Mesenchymal Stem Cell Behavior Under Microgravity: From Stress Response to a Premature Senescence. *Int. J. Mol. Sci.* 2023, *24*, 7753

**DOI:** 10.3390/ijms27198534

**Published:** 2026-09-24

**Authors:** Renzo Pala, Sara Cruciani, Alessia Manca, Giuseppe Garroni, Mohammed Amine EL Faqir, Veronica Lentini, Giampiero Capobianco, Antonella Pantaleo, Margherita Maioli

**Affiliations:** 1 Department of Biomedical Sciences, University of Sassari, Viale San Pietro 43/B, 07100 Sassari, Italy; renzopala6@gmail.com (R.P.); sara.cruciani@outlook.com (S.C.); alessia_manca@hotmail.it (A.M.); giugarroni21@gmail.com (G.G.); m.elfaqir@phd.uniss.it (M.A.E.F.); v.lentini@studenti.uniss.it (V.L.); apantaleo@uniss.it (A.P.); 2 Department of Medical, Surgical and Experimental Sciences, Gynecologic and Obstetric Clinic, University of Sassari, 07100 Sassari, Italy; capobia@uniss.it; 3 Center for Developmental Biology and Reprogramming (CEDEBIOR), Department of Biomedical Sciences, University of Sassari, Viale San Pietro 43/B, 07100 Sassari, Italy

In the original publication [1], there was a mistake in Figure 6 as published. An inadvertent technical error occurred during the final preparation, saving, or uploading of the revised figure file, which resulted in the Ctrl and 6h panels appearing identical in the published version. This was an unintentional figure-assembly error that affected the final panel composition. The corrected Figure 6 appears below. The authors state that the scientific conclusions are unaffected. This correction was approved by the Academic Editor. The original publication has also been updated.

## Figures and Tables

**Figure 6:**
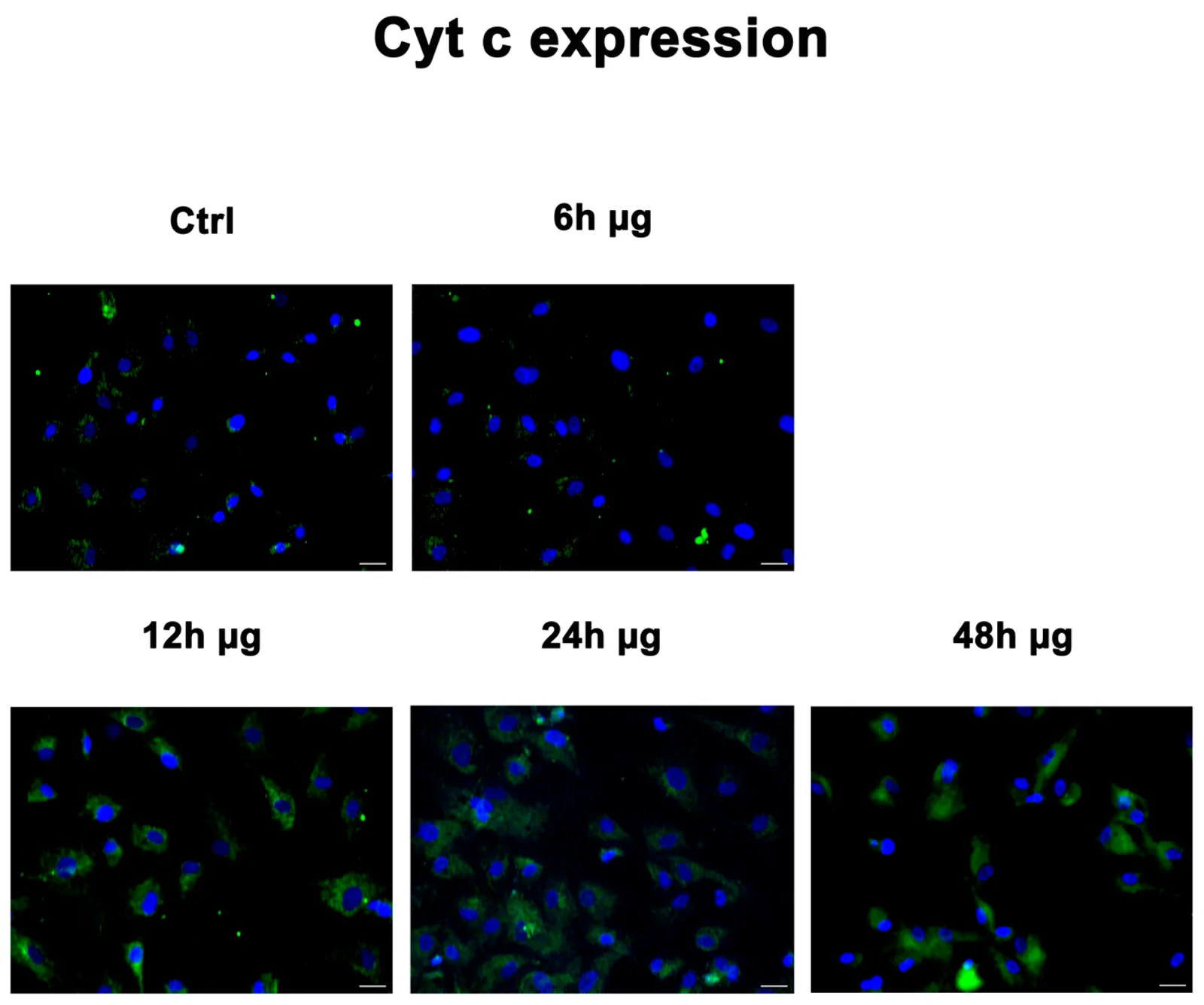
Analysis of Cyt C. Immunofluorescence analysis of Cyt C (green) was assessed in controls WJ-MSCs (Ctrl), and WJ-MSCs incubated from 6 to 48 h under microgravity conditions (µg). The figures are representative of different independent experiments. Nuclei are labelled with 4,6-diamidino-2-phenylindole (DAPI, blue). Magnification 40×. Scale bars: 40 µm.
